# Cognitive effects of untreated unilateral and bilateral hearing loss in midlife: Preliminary evidence

**DOI:** 10.1371/journal.pone.0361204

**Published:** 2026-10-21

**Authors:** Lakshmi Satheesan, Usha Shastri, Gagan Bajaj, Mohan Kumar Kalaiah

**Affiliations:** Department of Audiology and Speech Language Pathology, Kasturba Medical College Mangalore, Manipal Academy of Higher Education, Manipal, India; Cincinnati Children’s Hospital Medical Center, UNITED STATES OF AMERICA

## Abstract

Hearing loss has been identified as a modifiable risk factor for cognitive decline and dementia, although most of the evidence is derived from studies in older adults. Identifying such cognitive impairment in midlife is crucial, as it serves as a baseline for understanding changes in cognitive abilities that occur later in life. The present study aimed to compare the cognitive performance of age- and socioeconomic status-matched middle-aged adults with normal hearing and those with untreated hearing loss. This study included 48 participants aged 40–65 years, comprising 24 adults with normal hearing and 24 with untreated hearing loss. Among the latter participants, 9 had unilateral loss and 15 had bilateral loss. The cognitive evaluation involved assessing memory (forward and backward digit span and contextual memory test), attention via the Go/No-Go task, and executive function via the letter and category fluency tasks. Results showed that adults with untreated hearing loss performed significantly poorer on all the cognitive tasks compared to their normal-hearing counterparts. Large effect sizes were observed for memory tasks, the category fluency task and Go/No-Go accuracy, and a moderate effect size for the letter fluency tasks. Furthermore, multivariate linear regression analysis demonstrated that better-ear pure-tone thresholds independently predicted cognitive performance, but not age or education. These findings provide preliminary evidence on the association between untreated hearing loss and cognitive performance in middle age. However, considering the modest sample size and cross-sectional study design, longitudinal studies are needed to determine the temporal relationship between hearing loss and cognitive changes.

## Introduction

Midlife, typically spanning 40–65 years, represents far more than the midpoint of the lifespan [1,2]. It is a transitional stage in which emerging physiological, psychological and social changes begin to shape future health trajectories [3,4]. It serves as a baseline for understanding changes in cognitive abilities that occur later in life [5–7]. Given these multidimensional transitions, midlife represents a critical period during which emerging health conditions, particularly, sensory decline such as hearing loss may have disproportionate consequences for long-term cognitive and functional outcomes [8]. Acquired hearing loss in middle age can pose substantial challenges, particularly in complex listening environments encountered in occupational and social settings, such as workplace meetings, group discussions, and conversations involving multiple speakers [9,10]. Untreated hearing loss increases listening effort and fatigue, which can compromise occupational performance and contribute to psychosocial consequences, such as loneliness, social isolation, anxiety, and depression [11–14]. In addition to psychosocial effects, Xu et al. [15] highlighted possible alterations in the nervous, respiratory, psychiatric, and cardiometabolic systems associated with hearing loss during midlife. Among the 14 potentially modifiable risk factors identified by the 2024 Lancet Commission, midlife hearing loss was identified as one of the leading contributors to dementia risk, accounting for an estimated 7% of potentially preventable dementia cases worldwide [16]. These findings highlight the importance of timely identification and management of hearing loss across adulthood.

Epidemiological data from an Indian population-based study indicated that the prevalence of hearing loss increases with age, with an estimate of 4.1% among adults aged 40–49 years, 5.1% among those aged 50–59 years, and 21.8% among those aged 60–69 years [17]. Moreover, the ACHIEVE study reported that among older adults at increased risk for cognitive decline, hearing intervention slowed the loss of thinking and memory abilities by 48% over 3 years [18]. These findings highlight the potential importance of addressing hearing loss at an earlier age. This is particularly relevant to midlife, when cognitive decline may begin as young as 45–49 years of age [19]. As neuroplasticity and cognitive reserve remain relatively preserved during midlife, this stage represents an optimal window for interventions, where rehabilitation efforts may yield maximal benefit, by leveraging intact neural adaptability and compensatory mechanisms [2,5,7].

A recent systematic review and meta-analysis [20] revealed impairments in overall cognitive performance (Cohen’s d = −0.43) [21–26], memory (Cohen’s d = −0.25) [21–24] and executive function (Cohen’s d = −0.42) [21,22,24,25] in young and middle-aged adults with acquired hearing loss. Neuroimaging evidence has revealed greater volume declines in brain regions essential for cognition, such as the hippocampus and entorhinal cortex as a function of midlife hearing loss (45–65 years of age) [27]. These changes in brain structures closely mirror those observed in normal aging [28], suggesting a potential link between midlife hearing loss and adverse age-related changes in brain health. Hence, detecting those at risk of cognitive decline during middle age could be an important consideration in healthcare settings [29].

Hearing loss is a heterogeneous condition, and its cognitive consequences may vary according to characteristics of the impairment itself. In particular, laterality, duration of auditory deprivation, and treatment status may influence cognitive outcomes [18,23–25,30–39]. Existing studies have reported associations between both unilateral and bilateral hearing loss and poorer cognitive performance, although most have relied on global cognitive measures and have provided limited characterization of specific cognitive domains [30,31]. Moreover, hearing loss characteristics such as duration and treatment status are often inadequately documented, complicating the interpretation of observed cognitive differences [18,23–26,33–37,40]. Consequently, the cognitive effects of untreated hearing loss during midlife remain incompletely understood. In the present study, treatment status was restricted to untreated participants, laterality was examined by comparing unilateral and bilateral hearing loss, and the duration of hearing loss was documented but not included in the primary analyses.

Recent perspectives in audiology have emphasized age-related hearing loss as a potentially modifiable risk factor associated with cognitive impairment and have highlighted the importance of early identification of cognitive changes in individuals with hearing loss [41]. While the association between hearing loss and cognitive decline has been well-documented in older adults, limited evidence is available on whether middle-aged adults with untreated hearing loss demonstrate poorer performance across different cognitive domains than those with normal hearing, and whether cognitive performance differs between individuals with unilateral and bilateral hearing loss. Therefore, the present study adds to the literature by elucidating domain-specific cognitive performance in middle-aged adults with untreated hearing loss, while carefully matching participants for age and socioeconomic status.

## Method

### Participants

Approval for the current study was obtained from the Institutional Ethics Committee (IEC No: IEC KMC MLR 07/2024/468) before the recruitment of participants for the study. This study followed a standard group comparison research design conducted at a single time point between two groups: individuals with normal hearing (NH) and individuals with untreated hearing loss (HL). A 1:1 pair matching was performed on the basis of age and socio-economic status. There were 48 participants, with 24 in each group. Among the hearing loss participants, 9 had unilateral hearing loss (UHL), and 15 had bilateral hearing loss (BHL). Participants were recruited via convenience sampling from the Department of Audiology and Speech-Language Pathology at Kasturba Medical College, Mangalore. Prior to the study, written informed consent was obtained from the participants.

The participants were middle-aged adults with 40–65 years of age, in accordance with Sacco et al. [42]. All participants had at least 10 years of formal education to ensure basic literacy skills and adequate comprehension of visually presented stimuli and instructions. Cognitive screening was performed via the Montreal Cognitive Assessment (MoCA) [43,44], with a cutoff score of ≥26, to eliminate individuals with potential cognitive impairment. In addition, only those with a minimum rating of 7 (indicating good proficiency) for speaking and reading proficiency in the native language, as per the Language Experience and Proficiency Questionnaire (LEAP-Q) [45], were included. Group I consisted of individuals whose bilateral hearing thresholds were within normal limits (≤25 dB HL). Group II included individuals with untreated, postlingually acquired hearing loss, with the degree of hearing loss ranging from mild to severe. All types of hearing loss, including conductive, sensorineural, and mixed hearing loss, were considered with a minimum duration of hearing loss exceeding six months. Participants were excluded if they had a history of neurogenic or psychogenic conditions, uncorrected visual impairment, or congenital hearing loss, or if they were using amplification devices.

### Baseline assessment

Demographic and medical details, along with functional and disability-related information assessed using the WHO Ten Question Screener [46] were considered. Handedness was assessed via the Edinburgh Handedness Inventory (EHI) [47], and quality of life using the World Health Organization Quality of Life–Brief version (WHOQOL-BREF) scale [48]. In addition, socioeconomic status was determined using the modified Kuppuswamy socioeconomic scale [49,50] and interpreted by computing the total scores for education, occupation, and monthly income. Due to limited sample size, participants categorized as upper and upper middle class were combined into an upper socioeconomic status group, while those categorized as lower middle, upper lower and lower class were combined into a lower socioeconomic status group.

### Audiological assessment

Otoscopic examination was followed by Pure Tone Audiometry (0.25 kHz to 8 kHz) conducted in a sound-treated room via TDH-39 headphones and an AC40 diagnostic audiometer (Interacoustics, Middelfart, Denmark). Air- and bone-conduction thresholds were obtained separately for each ear via the Hughson—Westlake procedure [51]. Pure tone average (PTA) was calculated as the mean thresholds of 0.5 kHz, 1 kHz, 2 kHz, and 4 kHz frequencies, following ISO-7029 norms [52]. The degree of hearing loss was classified on the basis of Goodman’s criteria [53].

### Cognitive assessment

The cognitive assessment involved evaluating three core domains: memory, attention and executive function. The memory tests included the contextual memory test (CMT) [54], forward digit span (FDS) [55] and backward digit span (BDS) [55]. Attention was assessed via the Go/No-Go task [56], whereas executive functioning was evaluated via the verbal fluency task [57]. All the cognitive tasks, except the CMT, were administered via jsPsych version 8 [58]. CMT was presented separately via a timed PowerPoint presentation. For consistency and accessibility, all instructions and stimuli were presented visually on a personal laptop screen of 15.6 inches (Lenovo Ideapad 1 15ALC). Although these tasks were not linguistically demanding, language proficiency assessed using LEAP-Q ensured adequate comprehension of the task instructions and responses. Each task was preceded by a practice trial to ensure that the participants were familiar with the task structure and response method. The overall duration of the test was approximately 40 minutes. A rest period was provided as needed by the participant, and the task order was randomized across participants. When needed, the tasks were administered across two sessions, on the basis of the participant’s fatigue level. The details of the cognitive tests used in the study are given in the following subsection.

### Memory tests

Three tasks were performed to assess memory. All the tests were administered visually. Among them, CMT and FDS assess short-term memory, whereas BDS is used to assess working memory. The CMT was adapted from Toglia [54]. It is a functional cognitive assessment that has previously been used across diverse clinical and non-clinical populations [59,60]. The stimuli in the CMT consisted of 20 high-contrast images depicting a morning routine scene, including commonly encountered everyday objects such as bathroom, toothbrush, toothpaste, comb, and towels. The stimulus images were pre-tested on five individuals to ensure participant familiarity. All images were displayed simultaneously on a single PowerPoint slide for a fixed duration of 1.5 minutes. Participants were instructed to observe the images carefully and remember as many objects as possible during the 1.5-minute presentation. Following stimulus removal, a free recall phase was administered, during which participants verbally reported the objects they remembered. The scoring was based on the total number of correctly recalled items out of 20.

FDS and BDS were adapted from Jones and Macken [55]. In the FDS, participants recalled a string of digits in the same order in which they were presented, whereas in the BDS, the participants recalled a string of digits in reverse order. Stimuli were presented visually on a computer screen as Arabic numerals, with each digit displayed for 500ms, followed by a 250ms inter-stimulus interval. Participants verbally recalled the digits in their native language. Both digit spans employed a 1:2 staircase procedure, in which the span increased by one digit after a correct response, and decreased by one digit after two consecutive incorrect responses. The initial span length began at three digits. All participants completed the predetermined 12 trials, where the scoring was based on the highest span recalled correctly at least three times.

### Attention test

The Go/No-Go task, adapted from Mudar et al. [56], was used to evaluate selective attention. The participants were visually presented with black and white line drawings of a car (go stimuli) and a dog (no-go stimuli). They were instructed to press the space bar as quickly as possible when a car appeared on the screen, and to withhold responses when a dog was shown. The task consisted of 200 trials in total, with 160 trials and 40 No-Go trials. Each trial began with a fixation image displayed for 1700 ms, followed by the stimulus, which was presented for 300 ms. The total response window was 1000 ms from stimulus onset. Performance was assessed on the basis of accuracy and reaction time. Accuracy was calculated as the percentage of correct responses to Go stimuli and correct rejections to No-Go stimuli relative to the total number of trials. Reaction time was calculated as the time in milliseconds from stimulus onset to keypress for responses made within the response window. Trials without a response within this window were recorded as non-responses and were not assigned a reaction time value.

### Executive function test

Verbal fluency tasks (letter fluency task and category fluency task) were administered to assess executive function. In the letter fluency task, participants were instructed to generate as many words as possible within one minute, starting with the phonemes /k/ and /p/ in the native language of the participants. In the category fluency task, the participants were instructed to list as many words as possible within one minute that belonged to a semantic category (animals). The letter and the category used in the task were chosen on the basis of their frequency of occurrence and linguistic appropriateness within the native language [57]. Each task was timed for 60 seconds using the voice recorder application on a Samsung S24 FE smartphone, which provided a visible digital timer. Participants were able to see the timer during task performance. The responses were audio-recorded for subsequent transcription and scoring.

### Data analysis

Descriptive statistics were computed for demographic details, hearing and cognitive measures across two groups using an independent sample t-test and chi-square tests of association, where appropriate. Normality was assessed via the Shapiro-Wilk test, and for those measures (FDS, BDS and Go/No-go task) that did not follow a normal distribution, non-parametric tests were used. The Mann-Whitney U test was used to compare the cognitive performance between NH and HL participants, with p-values adjusted using Holm’s step-down method for multiple comparisons. For subgroup analysis, cognitive performance was compared among NH, UHL and BHL participants using the Kruskal-Wallis test. Dunn’s post hoc tests with Bonferroni adjustment were used for pairwise comparisons where appropriate.

Further, Spearman correlation analysis was conducted to examine the relationship between better ear PTA and cognitive performance, with Bonferroni adjustment for multiple comparisons. For this, the pooled data of the NH and HL groups were considered. To further examine the independent associations of hearing sensitivity with cognitive performance, a multivariate linear regression was performed using a composite cognitive score derived from principal component analysis (PCA) as the outcome variable and better ear PTA, age and education as predictors. The PCA-derived composite score was used to summarize the shared variance across the cognitive measures into a single global cognitive outcome. This approach reduced the number of dependent variables in the multivariate regression analysis and minimised the issue of multiple testing. Prior to PCA, the suitability of data for this analysis was indicated by a Kaiser-Meyer-Olkin (KMO) measure of sampling adequacy of 0.68, and a significant Barlett’s test of sphericity (p < 0.001). The analysis further indicated that a single principal component is sufficient to represent the cognitive measures, with an eigen value of 3.18, explaining 40% of the total variance and factor loadings ranging from 0.3 to 0.74.

The data were statistically analyzed via JASP (version 0.19.3) [61] and R software [62]. Statistical significance was set at p < 0.05. Given the exploratory nature of the study and the relatively modest sample size, findings were interpreted in conjunction with effect sizes, confidence intervals and consistency across cognitive domains rather than solely on the basis of statistical significance. The effect sizes were reported via the rank-biserial correlation (r) and eta-squared (η2) for the Mann-Whitney U tests and Kruskal-Wallis tests, respectively [63]. For Spearman’s correlation coefficient, the size of correlation was expressed as ρ [64].

## Results

Table 1 presents the demographic characteristics of the study participants. No significant between-group differences were observed in demographic, health, lifestyle, or quality-of-life variables (p > 0.05; S1 Table). Thus, the groups can be considered comparable in terms of demographic and clinical characteristics.

**Table 1:** Demographic characteristics of the study participants in the group with normal hearing (n = 24) and the group with hearing loss (n = 24).

Characteristics	Normal hearing	Hearing loss	
		Unilateral (n = 9)	Bilateral (n = 15)
**Age in years (Mean, SD)**	57.5 (7)	53.9 (8.01)	60 (5.21)
**Gender (n, %)**			
**Males**	10 (41.7%)	3 (33.3%)	9 (60%)
**Females**	14 (58.3%)	6 (66.7%)	6 (40%)
**Socioeconomic status (n, %)**			
**Lower class**	20 (83.3%)	8 (88.9%)	12 (80%)
**Upper class**	4 (16.7%)	1 (11.1%)	3 (20%)
**PTA in dB HL (Mean, SD)**			
**Better ear**	9.32 (5.71)	19.16 (7.2)	40.08 (13.29)
**Poorer ear**	12.69 (4.49)	39.44 (13.92)	52.02 (19.53)
**Duration of hearing loss in years** **Mean, SD** **Range**	–	2.86 (3.16) 0.6 - 10	7.76 (7.95) 0.8 - 20

Note: Upper class and upper middle class were categorized into “upper class” socioeconomic group whereas lower middle, upper lower and lower class were categorized into “lower class” socioeconomic group

### Cognitive performance across individuals with normal hearing and hearing loss

As depicted in Table 2, on comparing NH and HL, the Mann-Whitney U test revealed statistically significant differences with moderate to large effect sizes across all the cognitive tasks (p < 0.05), with the exception of the letter fluency task /k/ and Go/No-Go reaction time. The supplementary material (S2 Table) presents the mean, standard deviation and 95% confidence interval for each domain across the two groups.

**Table 2:** Median (Q1 and Q3) of cognitive performance across individuals with normal hearing and hearing loss and the results of the Mann-Whitney U test.

Cognitive tests	Normal Hearing	Hearing Loss	Z (p-value)	Effect size r (95% CI)
MoCA scores	27 (26,28)	27 (26,28)	243 (0.422)	0.158 (−0.866, 0.272)
**Memory**				
Forward digit span	6 (5,6)	5 (4,5)	456** (0.009)	0.583 (0.325, 0.761)
Backward digit span	5 (4,5)	4 (3,4)	462.5** (0.009)	0.606 (0.356, 0.775)
Contextual memory test	14 (13.5,16)	12 (10.5,13)	423** (0.009)	0.599 (0.341, 0.774)
**Executive function**				
Letter fluency /k/	14 (12,18)	13 (9,15.5)	348 (0.198)	0.318 (−0.008, 0.583)
Letter fluency /p/	15 (10, 18)	11 (7,13.5)	380* (0.044)	0.437 (0.130, 0.667)
Category fluency	18.5 (16,21.8)	14 (10.5,15.8)	376* (0.010)	0.556 (0.274, 0.749)
**Attention (Go/No-Go)**				
Accuracy (%)	89.5 (86, 94)	78.5 (71, 87)	354*** (0.009)	0.605 (0.335, 0.784)
Reaction time (ms)	397 (368,414)	420 (385,428)	170 (0.422)	−0.229 (−0.527, 0.120)

Note: p-values were adjusted using Holm’s step-down procedure. *p < 0.05, ** p < 0.01, *** p < 0.001; for the contextual memory test, the number of pictures recalled is provided; for letter fluency and category fluency, words/items per minute is provided.

### Relationship between hearing sensitivity and cognitive performance

Spearman’s correlation analyses conducted between better ear PTA and cognitive measures across the entire sample (Table 3) revealed a low to moderate, significant negative correlation between better ear PTA with other cognitive measures, except for letter fluency /k/ and Go/No-Go reaction time.

**Table 3:** Results of Spearman’s correlation analysis of the better ear PTA and cognitive test performances.

Variable	Cognitive domains	Cognitive tests	ρ	p-value
Better ear PTA	Memory	Forward digit span	−0.492	<0.001*
		Backward digit span	−0.536	<0.001*
		Contextual memory test	−0.482	<0.001*
	Executive function	Letter fluency /k/	−0.277	0.062
		Letter fluency /p/	−0.412	0.004*
		Category fluency	−0.517	<0.001*
	Attention	Accuracy (%)	−0.447	0.003*
		Reaction time (ms)	0.355	0.021

Note: *p < 0.006 is the adjusted significance threshold after applying Bonferroni correction across the eight cognitive outcome measures

A multivariate linear regression analysis was performed to further examine the effect of hearing sensitivity on overall cognitive performance, with PCA-derived composite cognitive score as the outcome variable and better ear PTA, age and education as predictors. The overall regression model revealed a statistically significant effect, explaining 39% of the variance in the composite cognitive measure [R^2^ = 0.39; F(3,36) = 7.89; p < 0.001]. Better-ear PTA emerged as the only significant independent predictor of composite cognitive performance (B = −0.038, p < 0.001), indicating that poorer hearing sensitivity was independently associated with lower cognitive scores (Table 4).

**Table 4:** Multivariate linear regression model predicting the effect of hearing sensitivity on PCA-derived composite cognitive score, adjusted for age and education.

Model fit metrics	Predictors	Estimate	SE	t value	p value
F(3,36) = 7.89	**Intercept**	0.991	1.516	0.654	0.517
p < 0.001	**Age**	−0.004	0.022	−0.214	0.831
R^2^ = 0.39	**Better ear PTA**	−0.038	0.009	−4.127	<0.001***
Adjusted R^2^ = 0.34	**Education**	0.020	0.121	0.165	0.870

Note: *p < 0.05, ** p < 0.01, *** p < 0.001; SE is the standard error

Table 5 depicts the comparison of cognitive performance across NH, UHL and BHL using the Kruskal-Wallis test. Although the omnibus Kruskal-Wallis analysis showed statistically significant differences across the NH, UHL and BHL groups for several cognitive tasks (p < 0.05), post hoc analyses indicate that the most significant effects were driven by differences between the NH and BHL groups. Comparisons involving the UHL group were generally non-significant, and no significant differences were observed between the UHL and BHL groups. Effect sizes ranged from small to large, with large effects observed for several memory and attention measures. The supplementary material (S1 Fig) presents raincloud plots depicting the cognitive performance across three groups. Additionally, the mean, standard deviation and 95% confidence interval for each domain across the three groups are presented in the supplementary material (S3 Table).

**Table 5:** Kruskal-Wallis test and Dunn’s Post Hoc comparisons of cognitive performance across individuals with normal hearing (NH), unilateral hearing loss (UHL) and bilateral hearing loss (BHL).

Cognitive tests	Kruskal-Wallis test			Dunn’s Post Hoc comparison	
	χ²	p-value	Effect size η2 (95% CI)	Comparisons	p-value
**Memory**					
Forward digit span	13.77	0.001**	0.261 (0.074, 0.527)	NH vs UHL NH vs BHL UHL vs BHL	0.011* 0.005** 1.000
Backward digit span	15.67	<0.001***	0.304 (0.150, 0.551)	NH vs UHL NH vs BHL UHL vs BHL	0.009** 0.002** 1.000
Contextual memory test	12.51	0.002**	0.244 (0.071, 0.517)	NH vs UHL NH vs BHL UHL vs BHL	0.057 0.003** 1.000
**Executive Function**					
Letter fluency /k/	3.49	0.175	0.035 (0, 0.266)	NH vs UHL NH vs BHL UHL vs BHL	0.357 0.416 1.000
Letter fluency /p/	6.49	0.039*	0.105 (0, 0.373)	NH vs UHL NH vs BHL UHL vs BHL	0.130 0.100 1.000
Category fluency	13	0.002**	0.268 (0.048, 0.509)	NH vs UHL NH vs BHL UHL vs BHL	0.755 <0.001*** 0.260
**Attention (Go/No-Go)**					
Accuracy (%)	13.98	<0.001***	0.307 (0.079, 0.593)	NH vs UHL NH vs BHL UHL vs BHL	0.927 <0.001*** 0.303
Reaction time (ms)	7.46	0.024*	0.140 (0, 0.392)	NH vs UHL NH vs BHL UHL vs BHL	0.339 0.095 0.072

Note: *p < 0.05, ** p < 0.01, *** p < 0.001; Bonferroni-adjusted p-values generated within JASP are reported for Dunn’s post hoc comparisons; for contextual memory test, the number of pictures recalled is provided; for letter fluency and category fluency, words/items per min is provided.

## Discussion

The findings from this preliminary analysis demonstrate that even in midlife, untreated hearing loss is significantly associated with poor performance across multiple cognitive domains, like memory, attention, and executive function, when compared with their normal hearing peers. Consistent with prior research that has focused predominantly on older adults [65,66], the current findings extend the evidence base by highlighting midlife as a critical stage for identifying early cognitive impairments linked to auditory deprivation.

Participants with HL showed significantly poorer performance across almost all cognitive tasks, namely the FDS, BDS, CMT, category fluency, and Go/No-Go tasks, with medium-to-large effect sizes. One possible explanation for these observed group differences may be the auditory deprivation hypothesis, which suggests that reduced auditory input diminishes stimulation to the brain regions that are responsible for cognitive processing [67]. In the present study, reaction time in the Go/No-Go task and letter fluency task /k/ did not reach statistical significance. The absence of significance in these tasks may be attributed to pre-existing group differences, task-specific demands, increased listening effort, restricted sampling, or unmeasured confounding factors.

The relationship between hearing sensitivity and cognitive performance was further supported by the multivariate regression model, which demonstrated that better-ear PTA independently predicted cognitive performance even after adjusting for age and education. This suggests that poorer hearing sensitivity may be associated with reduced cognitive performance beyond the effects of normal age or education related variations within the present sample. Although these findings should be interpreted within the context of the study design and sample characteristics, the results are consistent with previous literature demonstrating an association between greater hearing sensitivity and poor cognitive performance [68,69].

Exploratory subgroup comparisons showed that significant post hoc differences were predominantly observed between the NH and BHL groups, whereas comparisons involving the UHL group were generally not statistically significant. These findings provide limited evidence for differences in cognitive performance by hearing loss laterality and should be interpreted with caution, given the small and unequal subgroup sizes and the exploratory nature of the study.

Auditory-based cognitive tests may underestimate performance in individuals with hearing loss due to reduced audibility and increased listening fatigue [70,71] Hence, all the cognitive tasks and instructions in the present study were presented visually, thereby minimizing the confounding effects of audibility or speech perception difficulties and ensuring that the outcomes more accurately reflected cognitive abilities. These methodological considerations strengthen the preliminary findings of the study. The current findings further support recent perspectives emphasizing the important role of audiologists in the early identification of cognitive difficulties associated with hearing loss and the integration of cognitive considerations into routine audiological practice [41]. Furthermore, public health campaigns may benefit from focusing on increasing awareness of the cognitive risks associated with untreated hearing loss, even in the absence of subjective cognitive complaints.

### Limitation and future directions

Several limitations should be acknowledged. First, the relatively small sample size and unequal distribution of participants across the NH, UHL and BHL groups may have increased the risk of inflated findings and reduced the stability of subgroup comparisons. Hence, replication of these findings in future studies with larger and more evenly matched samples is needed to validate these preliminary observations. Second, the cross-sectional study design limits the ability to determine temporal relationships or infer progressive cognitive changes associated with hearing loss. Moreover, the use of convenience sampling along with a relatively high-functioning and homogeneous sample may have limited the broader generalizability of the findings. Therefore, future research must focus on longitudinal study designs to explore the trajectory of cognitive decline over time.

Third, the heterogeneity in the hearing loss characteristics of participants. Although sensitivity analyses excluding participants with greater severity (PTA above 55 dBHL in the better ear) and those with conductive hearing loss showed a similar pattern, strength and significance of results, these findings should be interpreted with caution and require replication in more homogeneous and larger samples. Additionally, the estimation of hearing loss duration was based on participants’ self-reports and potentially susceptible to recall bias. Henceforth, future studies should incorporate standardized and objective approaches for estimating hearing loss characteristics.

Fourth, the limited standardization of certain cognitive measures, particularly the verbal fluency tasks and CMT. The verbal fluency performance may have been influenced not only by executive functioning but also by lexical retrieval and linguistic factors. In addition, although the phonemes selected for the verbal fluency tasks were chosen based on their linguistic appropriateness and frequency within the native language, the use of a limited number of phoneme-based fluency tasks may restrict comparability with studies employing broader standardized verbal fluency batteries. Similarly, although CMT is an established functional cognitive assessment used across diverse population, it had limited large-scale standardization within the target population, which might have affected the findings. Future studies should incorporate broader standardization and validation within the target population to improve reliability and generalizability of the findings.

## Conclusion

The current study adds to the growing body of evidence linking midlife untreated hearing loss with early cognitive impairment. Middle-aged adults with untreated hearing loss demonstrated poorer performance across multiple cognitive domains when compared with individuals with normal hearing. These findings highlight the importance of considering cognitive functioning during audiological evaluation and rehabilitation. Although the current study provides preliminary evidence on the association between untreated hearing loss and cognitive performance, these findings should be interpreted cautiously, given the cross-sectional design and modest sample size. Longitudinal studies with larger and more representative samples are needed to determine the temporal relationship between hearing loss and cognitive changes.

## Supporting information

S1 Table Distribution of additional demographic and clinical characteristics across groups and their statistical significance using the Chi-square test. (DOCX)

S2 Table Distribution of mean, standard deviation and 95% confidence interval across individuals with normal hearing and hearing loss in different cognitive domains. (DOCX)

S1 Fig (a). Raincloud plot depicting the performance of individuals with normal hearing, unilateral hearing loss and bilateral hearing loss in the memory domain. (b). Raincloud plot depicting the performance of individuals with normal hearing, unilateral hearing loss and bilateral hearing loss in the executive function domain. (c). Raincloud plot depicting the performance of individuals with normal hearing, unilateral hearing loss and bilateral hearing loss in the attention domain.
(DOCX)

S3 Table Distribution of mean, standard deviation and 95% confidence interval across individuals with normal hearing, unilateral hearing loss and bilateral hearing loss in different cognitive domains. (DOCX)

S1 File Raw data of participants showing distribution of age, gender, socioeconomic status, PTA, type, degree and duration of hearing loss with corresponding cognitive outcomes. (XLSX)
