# Supplementary material for: Cognitive effects of untreated unilateral and bilateral hearing loss in midlife: Preliminary evidence
Source: PLoS One. 2026 Oct 21;21(10):e0361204. doi: 10.1371/journal.pone.0361204 (PMC13686519; doi:10.1371/journal.pone.0361204)
Supplement: Supplementary file 1 [file pone.0361204.s001.docx]

**S1 Table. Distribution of additional demographic and clinical characteristics across groups and their statistical significance using the Chi-square test**

| **Variables** | **Normal Hearing** | **Hearing loss** | **p value** |
| --- | --- | --- | --- |
| **Hypertension** | | | |
| **Yes** | 11 (45.8%) | 10 (41.7%) | 0.771 |
| **No** | 13 (54.2%) | 14 (58.3%) |  |
| **Diabetes** | | | |
| **Yes** | 7 (29.2%) | 6 (25%) | 0.745 |
| **No** | 17 (70.8%) | 18 (75%) |  |
| **Heart diseases** | | | |
| **Yes** | 4 (16.7%) | 4 (16.7%) | 1 |
| **No** | 20 (83.3%) | 20 (83.3%) |  |
| **High cholesterol** | | | |
| **Yes** | 6 (25%) | 6 (25%) | 1 |
| **No** | 18 (75%) | 18 (75%) |  |
| **Thyroid problems** | | | |
| **Yes** | 2 (8.3%) | 1 (4.2%) | 1 |
| **No** | 22 (91.7%) | 23 (95.8%) |  |
| **Kidney diseases** | | | |
| **Yes** | 3 (12.5%) | 1 (4.2%) | 0.296 |
| **No** | 21 (87.5%) | 23 (95.8%) |  |
| **Liver diseases** | | | |
| **Yes** | 0 (0%) | 1 (4.2%) | 1 |
| **No** | 24 (100%) | 23 (95.8%) |  |
| **Joint problems** | | | |
| **Yes** | 4 (16.7%) | 4 (16.7%) | 1 |
| **No** | 20 (83.3%) | 20 (83.3%) |  |
| **Exposure to loud noise** | | | |
| **Yes** | 1 (4.2%) | 4 (16.7%) | 0.348 |
| **No** | 23 (95.8%) | 20 (83.3%) |  |
| **Physical activity** | | | |
| **Sedentary** | 5 (20.8%) | 6 (25%) | 0.267 |
| **Moderate** | 17 (70.9%) | 12 (50%) |  |
| **High** | 2 (8.3%) | 6 (25%) |  |
| **Smoking** | | | |
| **Never** | 22 (91.7%) | 16 (66.7%) | 0.072 |
| **Past smoker** | 2 (8.3%) | 7 (29.1%) |  |
| **Current smoker** | 0 (0%) | 1 (4.2%) |  |
| **Alcohol use** | | | |
| **Yes** | 6 (25%) | 9 (37.5%) | 0.350 |
| **No** | 18 (75%) | 15 (62.5%) |  |
| **Physical quality of life** | | | |
| **Very poor** | 0 (0%) | 0 (0%) | 0.350 |
| **Poor** | 0 (0%) | 0 (0%) |  |
| **Moderate** | 0 (0%) | 0 (0%) |  |
| **Good** | 6 (25%) | 9 (37.5%) |  |
| **Very good** | 18 (75%) | 15 (62.5%) |  |
| **Psychological quality of life** | | | |
| **Very poor** | 0 (0%) | 0 (0%) | 0.182 |
| **Poor** | 0 (0%) | 0 (0%) |  |
| **Moderate** | 0 (0%) | 0 (0%) |  |
| **Good** | 4 (16.7%) | 8 (33.3%) |  |
| **Very good** | 20 (83.3%) | 16 (66.7%) |  |
| **Environmental quality of life** | | | |
| **Very poor** | 0 (0%) | 0 (0%) | 0.755 |
| **Poor** | 0 (0%) | 0 (0%) |  |
| **Moderate** | 0 (0%) | 0 (0%) |  |
| **Good** | 7 (29.3%) | 8 (33.2%) |  |
| **Very good** | 16 (66.7%) | 17 (70.8%) |  |
| **Social quality of life** | | | |
| **Very poor** | 0 (0%) | 0 (0%) | 1 |
| **Poor** | 0 (0%) | 0 (0%) |  |
| **Moderate** | 0 (0%) | 0 (0%) |  |
| **Good** | 5 (20.8%) | 5 (20.8%) |  |
| **Very good** | 19 (79.2%) | 19 (79.2%) |  |
| **Handedness** | | | 0.312 |
| **Right handers** | 24 (100%) | 23 (95.8%) |  |
| **Mixed handers** | 0 (0%) | 1 (4.2%) |  |
