# Supplementary material for: Cognitive effects of untreated unilateral and bilateral hearing loss in midlife: Preliminary evidence
Source: PLoS One. 2026 Oct 21;21(10):e0361204. doi: 10.1371/journal.pone.0361204 (PMC13686519; doi:10.1371/journal.pone.0361204)
Supplement: Supplementary file 2 [file pone.0361204.s002.docx]

**S2 Table. Distribution of mean, standard deviation and 95% confidence interval across individuals with normal hearing and hearing loss in different cognitive domains**

| **Cognitive tests** | **Normal hearing**  **Mean, SD**  **(95% CI)** | **Hearing loss**  **Mean, SD**  **(95% CI)** |
| --- | --- | --- |
| **MoCA** | 27.208 ± 1.021  (26.77, 27.63) | 26.917 ± 0.929  (26.52, 27.30) |
| **Memory** | | |
| Forward digit span | 5.87 ± 0.992  (5.45, 6.29) | 4.79 ± 0.779  (4.46, 5.12) |
| Backward digit span | 4.79 ± 0.93  (4.39, 5.18) | 3.79 ± 0.58  (3.54,4.04) |
| Contextual memory test | 14 .65 ± 2.22 (13.68,15.61) | 11.69 ± 2.67 (10.54,12.85) |
| **Executive function** | | |
| Letter fluency /k/ | 14.91 ± 4.91  (12.78,17.04) | 12.17 ± 4.67  (10.15,14.19) |
| Letter fluency /p/ | 14.04 ± 4.28  (12.19, 15.89) | 10.26 ± 4.4  (8.36, 12.16) |
| Category fluency | 18.31 ± 4.41  (16.36, 20.27) | 13.81 ± 4.27  (11.92, 15.71) |
| **Attention** | | |
| Accuracy (%) | 88.28 ± 7.84  (84.71, 91.85) | 77.45 ± 11.43  (72.24, 82.65) |
| Reaction time (ms) | 395.96 ± 43.89  (375.98, 415.94) | 408.16 ± 37.61 (391.04,425.28) |

Note: For the contextual memory test, the number of pictures recalled is provided; for letter fluency and category fluency, the words/items per min is provided.
