# Supplementary material for: Cognitive effects of untreated unilateral and bilateral hearing loss in midlife: Preliminary evidence
Source: PLoS One. 2026 Oct 21;21(10):e0361204. doi: 10.1371/journal.pone.0361204 (PMC13686519; doi:10.1371/journal.pone.0361204)
Supplement: Supplementary file 3 [file pone.0361204.s003.docx]

**Fig S3 (a). Raincloud plot depicting the performance of individuals with normal hearing, unilateral hearing loss and bilateral hearing loss in the memory domain**

Note: Normal hearing (NH), unilateral hearing loss (UHL), bilateral hearing loss (BHL). For each test dataset, within each group, the first panel displays the distribution of the data, the box plot shows the median and interquartile range (the diamond in the box plot indicates the mean), and the dots represent the individual data points

**Fig S3 (b). Raincloud plot depicting the performance of individuals with normal hearing, unilateral hearing loss and bilateral hearing loss in the executive function domain**

Note: Normal hearing (NH), unilateral hearing loss (UHL), bilateral hearing loss (BHL). For each test dataset, within each group, the first panel displays the distribution of the data, the box plot shows the median and interquartile range (the diamond in the box plot indicates the mean), and the dots represent the individual data points.

**Fig S3 (c). Raincloud plot depicting the performance of individuals with normal hearing, unilateral hearing loss and bilateral hearing loss in the attention domain**

Note: Normal hearing (NH), unilateral hearing loss (UHL), bilateral hearing loss (BHL). For each test dataset, within each group, the first panel displays the distribution of the data, the box plot shows the median and interquartile range (the diamond in the box plot indicates the mean), and the dots represent the individual data points
