# Supplementary material for: Cognitive effects of untreated unilateral and bilateral hearing loss in midlife: Preliminary evidence
Source: PLoS One. 2026 Oct 21;21(10):e0361204. doi: 10.1371/journal.pone.0361204 (PMC13686519; doi:10.1371/journal.pone.0361204)
Supplement: Supplementary file 4 [file pone.0361204.s004.docx]

**S4 Table. Distribution of mean, standard deviation and 95% confidence interval across individuals with normal hearing, unilateral hearing loss and bilateral hearing loss in different cognitive domains**

| **Cognitive tests** | **Normal hearing**  **Mean, SD**  **(95% CI)** | **Unilateral hearing loss**  **Mean, SD**  **(95% CI)** | **Bilateral hearing loss**  **Mean, SD**  **(95% CI)** |
| --- | --- | --- | --- |
| **Memory** | | | |
| Forward digit span | 5.87 ± 0.992  (5.45, 6.29) | 4.66 ± 1  (3.89, 5.43) | 4.86 ± 0.640  (4.51, 5.22) |
| Backward digit span | 4.79 ± 0.93  (4.39, 5.18) | 3.77 ± 0.66  (3.26,4.29) | 3.8 ± 0.56  (3.49,4.11) |
| Contextual memory test | 14 .65 ± 2.22 (13.68,15.61) | 11.88 ± 3.18 (9.44,14.33) | 11.57 ± 2.40 (10.18,12.96) |
| **Executive function** | | | |
| Letter fluency /k/ | 14.91 ± 4.91  (12.78,17.04) | 11.77 ± 4.29  (8.47,15.07) | 12.42 ± 5.03  (9.52,15.33) |
| Letter fluency /p/ | 14.04 ± 4.28  (12.19, 15.89) | 10.22 ± 4.2  (6.98, 13.45) | 10.28 ± 4.68  (7.58, 12.98) |
| Category fluency | 18.31 ± 4.41  (16.36, 20.27) | 16.12 ± 4.12  (12.68, 19.57) | 12.5 ± 3.89  (10.25, 14.75) |
| **Attention** | | | |
| Accuracy (%) | 88.28 ± 7.84  (84.71, 91.85) | 84 ± 8.56  (75, 92.99) | 74.83 ± 11.6  (68.40, 81.26) |
| Reaction time (ms) | 395.96 ± 43.89  (375.98, 415.94) | 377.14 ± 35.68 (339.69,414.58) | 420.56 ± 31.44 (403.15,437.98) |

Note: For the contextual memory test number of pictures recalled is provided; for letter fluency and category fluency, words/items per min is provided
